# Paradoxical Insomnia in the Presence of Mild Obstructive Sleep Apnea: A Case Report Illustrating Diagnostic Complexity and Treatment Challenges

**DOI:** 10.7759/cureus.100161

**Published:** 2025-12-26

**Authors:** Michael Goldberg, Avram Warshawsky, Suman Jalan

**Affiliations:** 1 Psychiatry, New York Medical College, Boonton, USA; 2 Psychiatry, Touro College of Osteopathic Medicine, New York, USA; 3 Psychiatry, Saint Clare’s Hospital, Boonton, USA

**Keywords:** case report, cognitive behavioral therapy for insomnia, insomnia, obstructive sleep apnea, paradoxical insomnia, sleep-state misperception

## Abstract

Paradoxical insomnia (sleep-state misperception) is characterized by a marked discrepancy between self-reported poor sleep and objective polysomnographic evidence of normal sleep duration and architecture. We present a case of paradoxical insomnia comorbid with mild obstructive sleep apnea (OSA), highlighting diagnostic and therapeutic challenges. A 37-year-old woman presented with severe chronic insomnia of eight years’ duration, reporting sleeping only "minutes per night," despite appearing alert and functional. A two-night home sleep test showed normal sleep latency (seven minutes), sleep efficiency (86.6%), and total sleep time (4.5 hours). Mild OSA was noted (Apnea-Hypopnea Index (AHI) = 5 events/hour) but was not considered causative of her profound subjective distress. Multiple trials of hypnotics, antidepressants, benzodiazepines, and antipsychotics yielded no subjective improvement. The patient maintained a fixed, delusional-like conviction of sleeplessness despite objective evidence to the contrary, although she lacked other psychotic features. This case underscores the importance of recognizing paradoxical insomnia, particularly when complaints are disproportionate to objective findings and mild comorbid OSA is present. Management should prioritize cognitive behavioral therapy for insomnia (CBT-I) and psychoeducation over pharmacologic escalation. Clinicians should communicate objective sleep data with empathy within a psychoeducational framework to avoid therapeutic rupture and promote engagement in behavioral interventions.

## Introduction

Insomnia is a common disorder, affecting an estimated 6%-33% of the general population [1,2]. A subset of patients presents with paradoxical insomnia (also termed sleep-state misperception), a clinical descriptor within the broader diagnosis of Insomnia Disorder ((Diagnostic and Statistical Manual of Mental Disorders, Fifth Edition (DSM-5), International Classification of Diseases, 11th Revision (ICD-11)) [3,4]. It is defined by a striking mismatch between subjective complaints of severe sleeplessness and objective evidence of relatively normal sleep duration and architecture [5,6]. Emerging evidence suggests that paradoxical insomnia may involve altered sleep microstructure, hyperarousal, or neurocognitive abnormalities during sleep rather than a quantitative reduction in sleep time [7,8]. Comorbid psychiatric conditions, particularly anxiety and obsessive-compulsive traits, are common and may exacerbate the misperception. The diagnostic picture can be further complicated by comorbid sleep-disordered breathing, such as obstructive sleep apnea (OSA), a condition characterized by repeated episodes of partial or complete upper airway obstruction during sleep, quantified by the Apnea-Hypopnea Index (AHI) [9]. Differentiating paradoxical insomnia from other insomnia subtypes and comorbid conditions is critical to avoid unnecessary pharmacologic interventions and to direct patients toward evidence-based behavioral treatments, such as cognitive behavioral therapy for insomnia (CBT-I).

## Case presentation

A 37-year-old woman presented with an eight-year history of severe, chronic insomnia, persistently reporting sleeping only "minutes per night." She described significant difficulty with both sleep initiation and maintenance, accompanied by intrusive ruminations about sleep loss and daytime anxiety regarding nighttime sleep. Despite appearing alert and functional, she scored 2 on the Epworth Sleepiness Scale (ESS) [10] and 28 on the Insomnia Severity Index (ISI) [11], indicating severe clinical insomnia in the absence of significant daytime sleepiness. A two-night home sleep test (acknowledging its limitations in assessing sleep microstructure and perception) revealed normal sleep architecture with mild OSA (Apnea-Hypopnea Index (AHI) = 5 events/hour), objectively confirming over 4.5 hours of sleep. Multiple pharmacological trials, including hypnotics, sedating antidepressants, benzodiazepines, and antipsychotics, provided no subjective improvement (Table 1). The patient maintained a fixed belief of sleeplessness despite contrary evidence. The ESS and ISI results are summarized in Tables 2, 3, respectively.

**Table 1:** Summary of Previous Pharmacological Interventions

Medication Class	Specific Medication and Regimen	Duration	Reported Outcome
Non-benzodiazepine hypnotics	Zolpidem 10 mg nightly	2 nights	No subjective benefit
	Eszopiclone 2 mg nightly	2 nights	No subjective benefit
Sedating antidepressants	Trazodone up to 150 mg nightly	~3 weeks	Minimal change
	Doxepin 10 mg nightly	2 nights	Minimal change
	Mirtazapine 15 mg nightly	~3 weeks	No perceived sleep
Benzodiazepines	Lorazepam 1-2 mg nightly	3 nights	No benefit reported
	Temazepam 15 mg nightly	2 nights	No benefit reported
Antipsychotics	Quetiapine 25-50 mg nightly	2 nights	No subjective improvement
	Olanzapine 5 mg nightly	2 nights	No subjective improvement
	Risperidone 1 mg nightly	1 night	No subjective improvement
	Lurasidone 20 mg nightly	1 night	No subjective improvement
Other sedatives	Hydroxyzine 50 mg nightly	2 nights	No reported sleep
	Gabapentin 300 mg nightly	2 nights	No reported sleep

**Table 2:** Epworth Sleepiness Scale (ESS) Results Note: A score ≤10 is considered normal [8].

Situation	Score (0-3)
Sitting and reading	0
Watching television	0
Sitting inactive in a public place	0
Being a passenger in a car for an hour	1
Lying down to rest in the afternoon	0
Sitting and talking to someone	0
Sitting quietly after lunch	0
Stopped in traffic while driving	1
Total score	2

**Table 3:** Insomnia Severity Index (ISI) Results Note: Scores 22-28 indicate severe clinical insomnia [9].

ISI Item	Score (0-4)
1. Severity of sleep onset difficulty	4
2. Severity of sleep maintenance difficulty	4
3. Severity of early morning awakening	4
4. Dissatisfaction with sleep pattern	4
5. Noticeability of impairment	4
6. Worry/Distress about sleep problem	4
7. Interference with daily functioning	4
Total score	28

Investigations

A two-night home sleep test was performed. The key objective findings and their clinical interpretation are summarized in Table 4. These findings objectively confirmed adequate sleep duration and architecture, with only mild, non-positional OSA present. When presented with the results, the patient expressed profound disbelief, insisting that the device was inaccurate and maintaining that she had been awake “all night.”

**Table 4:** Home Sleep Test Results and Interpretation SpO₂: peripheral capillary oxygen saturation.

Parameter	Result	Interpretation
Sleep latency	7 minutes	Normal sleep onset
Total sleep time	4.49 hours	Objectively confirms substantial sleep duration (>4 hours)
Sleep efficiency	86.6%	Good sleep continuity (normal >85%)
Mean SpO₂	95.9%	Normal oxygen saturation
Lowest SpO₂	No desaturation below 90%	Absence of significant hypoxemia
Apnea-Hypopnea Index (AHI)	5.0 events/hour	Meets criteria for mild obstructive sleep apnea (OSA) [7]
Supine sleep	63.7%	Indicates positional sleep but not predominantly supine

Differential diagnosis

The clinical presentation prompted consideration of several diagnostic possibilities. The profound and persistent mismatch between the patient's subjective conviction of near-total sleeplessness and the objective evidence of normal sleep architecture and duration, coupled with refractoriness to multiple classes of sedating medications, was most consistent with a primary diagnosis of insomnia disorder with features of paradoxical insomnia (sleep-state misperception). This perceptual disorder was likely exacerbated by anxiety-related hypervigilance and a somatic misperception, where quiet wakefulness or light sleep was misinterpreted as full wakefulness. An obsessive focus on sleep, characterized by intrusive ruminations about not sleeping, was a prominent perpetuating factor. While mild obstructive sleep apnea was objectively present, its severity (AHI=5) was insufficient to explain the degree of subjective distress or the reported complete lack of sleep, making it a comorbid condition rather than the primary etiology. Notably, polysomnography is considered more reliable than home testing for diagnosing mild OSA, as home studies can underestimate AHI [12,13]. Generalized Anxiety Disorder (GAD) and Obsessive-Compulsive Disorder (OCD) were considered as differentials given the somatic preoccupation and intrusive thoughts; however, the patient’s anxiety and obsessive thoughts were exclusively centered on sleep, not meeting full criteria for GAD or OCD. A somatic delusional disorder was considered but deemed less likely due to the patient's preserved insight in other domains, absence of other psychotic features, and the primary emotional distress centered on the sleep complaint itself rather than a fixed, bizarre somatic belief.

Treatment and outcome

Given the failure of pharmacotherapy, treatment shifted to non-pharmacological intervention. A structured cognitive behavioral therapy for insomnia (CBT-I) program was recommended, focusing on sleep restriction, stimulus control, cognitive restructuring, and relaxation training. Psychoeducation regarding paradoxical insomnia and the home sleep test results was provided repeatedly. Over six weeks of follow-up, the patient began to reluctantly reframe brief sleep episodes as “light rest,” although her core conviction of being awake most of the night persisted. Due to ongoing distress and limited insight, she was referred to a residential sleep-disorder behavioral program for intensive intervention.

## Discussion

This case illustrates the diagnostic complexity that arises when paradoxical insomnia co-occurs with mild OSA. While the presence of mild OSA can sometimes explain or exacerbate sleep complaints, its clinical profile in this case was incongruent with the patient's extreme report of total sleeplessness. The core pathology aligned more closely with the hypervigilance, somatic focus, and catastrophic misinterpretation characteristic of paradoxical insomnia, as described in the literature [5,6]. This reinforces the principle that comorbid conditions must be carefully evaluated for their causal contribution to the primary complaint; in this case, mild OSA was a comorbid finding, not the primary driver of the profound sleep misperception.

The patient's complete lack of response to multiple drug classes, spanning traditional hypnotics to sedating antipsychotics, is a hallmark of paradoxical insomnia and underscores that it is generally refractory to standard hypnotic pharmacotherapy [6,14]. As medications that increase sleep drive do not correct the underlying perceptual distortion, this case powerfully reinforces the established guideline that first-line management for chronic insomnia, and particularly its paradoxical subtype, should be CBT-I, which directly targets the maladaptive thoughts and behaviors perpetuating the condition [6,15].

A significant clinical challenge encountered was the communication of objective sleep data. For the patient, being told she had "slept several hours" was perceived as invalidating and paradoxically increased her anxiety, a known risk that can lead to therapeutic rupture [16]. This experience highlights that clinicians must deliver such feedback with profound empathy and within a robust psychoeducational framework, normalizing the phenomenon of sleep-state misperception to foster engagement rather than defensiveness [6,16].

This case underscores several critical clinical lessons. First, paradoxical insomnia should be actively considered when a patient presents with severe subjective complaints that are strikingly disproportionate to objective sleep findings. Second, the presence of a mild comorbid condition such as OSA does not preclude a primary diagnosis of paradoxical insomnia and requires careful, nuanced clinical interpretation to avoid attributing the core complaint to an incidental finding. Third, pharmacotherapy alone is often ineffective for paradoxical insomnia, as medications do not correct the underlying perceptual distortion; therefore, first-line management should center on cognitive behavioral therapy for insomnia (CBT-I) and targeted psychoeducation. Finally, communicating objective sleep results to the patient demands sensitivity and must be framed within a supportive therapeutic alliance to avoid invalidating the patient's experience, which could otherwise lead to disengagement and therapeutic rupture.

Limitations

This report is based on a single case. Home sleep testing, while useful, has limitations in assessing sleep microstructure and perception-related phenomena compared to in-lab polysomnography. Furthermore, the evolving diagnostic classification of paradoxical insomnia within broader insomnia disorder categories should be considered in clinical practice. Future research could explore whether repeated apnea events may trigger hyperarousal states contributing to chronic insomnia symptoms and identify biomarkers predictive of concurrent OSA and paradoxical insomnia.

## Conclusions

This case report highlights the critical importance of recognizing paradoxical insomnia, a condition defined by a profound subjective-objective sleep discrepancy. Accurate diagnosis is essential to avoid iatrogenic harm from escalating and ineffective pharmacotherapy and to steer patients toward evidence-based behavioral interventions. The co-occurrence of mild OSA adds a layer of diagnostic complexity but does not negate the primary perceptual pathology. The key message is that management must prioritize cognitive behavioral therapy for insomnia and careful, empathetic psychoeducation over pharmacological strategies. Future clinical approaches should integrate objective sleep testing with a psychologically informed framework to effectively diagnose and treat this challenging condition.
